# Supplementary material for: A computational model of pig ventricular cardiomyocyte electrophysiology and calcium handling: Translation from pig to human electrophysiology
Source: PLoS Comput Biol. 2021 Jun 30;17(6):e1009137. doi: 10.1371/journal.pcbi.1009137 (PMC8277015; doi:10.1371/journal.pcbi.1009137)
Supplement: S1 Text — (PDF) [file pcbi.1009137.s001.pdf]

S1 Text

Table of Contents

Table of Contents 1

DEFINITIONS AND ABBREVIATIONS 3

Cell Geometry 3

Currents 3

Gates 4

Conductances and Permeability 5

Concentrations 5

Buffers 7

Reversal Potentials 7

Others 7

MODEL PARAMETERS 8

Cell Geometry 8

Fixed Ionic Concentrations 9

Initial Conditions (at CL=1000 ms) 9

Physical Constants 10

Reversal Potentials 10

MODEL ELECTROPHYSIOLOGY 10

Fast Sodium Current (I_{Na}) 10

Late Sodium Current (I_{NaL}) 11

L-type Current 11

Rapid component of delayed rectifier K^+ current (I_{Kr}) 13

Slow component of delayed rectifier K^+ current (I_{Ks}) 13

Inward rectifier K^+ current (I_{K1}) 13

Ca^{2+}-activated Cl^- current (I_{to2}) 14

Na^+-Ca^{2+} exchanger current (I_{NaCa}) 14

Na^+- K^+ ATPase current (I_{NaK}) 14

Background K^+ current (I_{Kb}) 15

Background Na^+ current (I_{Nab}) 15

Background Ca^{2+} current (I_{Cab}) 15
Sarcolemmal Ca^{2+} pump current (I_{pCa}) 15
MODEL Membrane voltage (V_m) 15
MODEL Ca^{2+} handling 15
Ca^{2+}/Calmodulin-dependent Protein Kinase (CaMK) 15
SR Ca^{2+} SERCA/PLB Uptake flux 17
SR Ca^{2+} release from subspace in t-tubular region 17
SR Ca^{2+} release in the non t-tubular region 18
Spontaneous Overload Induced SR Ca^{2+} release (SOICR) by Ryanodine Receptors (J_{relol}) 19
Ca^{2+} concentrations in myoplasm, subspace, network SR, junctional SR and corbular SR 19
MODEL Na^+ handling 20
Na^+ concentration in the subspace and myoplasm 20
MODEL K^+ handling 20
K^+ concentrations in the subspace and myoplasm 20
Table S1: MODEL parameters and formulation sources. 22
References 25

DEFINITIONS AND ABBREVIATIONS

Cell Geometry

L_{cell}	Length of the cell (mm)
R_{cell}	Radius of the cell (mm)
V_{cell}	Volume of the cell (μL)
A_{geo}	Geometric area of the cell (cm^2)
A_{cap}	Capacitive area of the cell (cm^2)
V_{myo}	Volume of the myoplasm (μL)
V_{myo1}	Volume of the myoplasm in the t-tubular region (μL)
V_{myo2}	Volume of the myoplasm in the non t-tubular region (μL)
V_{NSR}	Volume of the network sarcolplasmic reticulum (NSR) (μL)
V_{NSR1}	Volume of NSR in the t-tubular region (μL)
V_{NSR2}	Volume of NSR in the non t-tubular region (μL)
V_{JSR}	Volume of the junctional sarcoplasmic reticulum (JSR) (μL)
V_{CSR}	Volume of the corbular sarcoplasmic reticulum (CSR) (μL)
V_{ss}	Volume of the subspace (μL)

Currents

I_{Na}	Fast sodium (Na^+) current (μA/μF)
I_{NaL}	Late sodium (Na^+) current (μA/μF)
I_{CaL}	Ca^{2+} current through L-type channels (μA/μF)
I_{CaNa}	Na^+ current through L-type channels (μA/μF)
I_{CaK}	K^+ current through L-type channels (μA/μF)
I_{Kr}	Rapid component of the delayed rectifier K^+ current (μA/μF)
I_{Ks}	Slow component of the delayed rectifier K^+ current (μA/μF)
I_{K1}	Inward rectifier K^+ current (μA/μF)
I_{to2}	Transient outward Ca^{2+} activated Cl^- current (μA/μF)

I_{NaCai}	Na^+-Ca^{2+} exchanger current in the myoplasm (μA/μF)
I_{NaCass}	Na^+-Ca^{2+} exchanger current in the subspace (μA/μF)
I_{NaK}	Na^+-K^+ ATPase pump current (μA/μF)
I_{Nab}	Background Na^+ current (μA/μF)
I_{Cab}	Background Ca^{2+} current (μA/μF)
I_{Kb}	Background K^+ current (μA/μF)
I_{pCa}	Sarcolemmal Ca^{2+} pump current (μA/μF)

Gates

m	Voltage-activation gate for I_{Na}
h	Fast voltage-inactivation gate for I_{Na}
j	Slow voltage-inactivation gate for I_{Na}
ml	Voltage-activation gate for I_{NaL}
hl	Voltage-inactivation gate for I_{NaL}
d	Voltage-activation gate for I_{CaL}
f	Voltage-inactivation gate for I_{CaL}
ff	Fast voltage-inactivation gate for I_{CaL}
fs	Slow voltage-inactivation gate for I_{CaL}
f_{Ca}	Ca^{2+}-inactivation gate for I_{CaL}
x_r	Voltage-activation gate for I_{Kr}
r_{kr}	Voltage-rectifier gate for I_{Kr}
x_{s1}	Voltage-activation gate for I_{Ks}
x_{s2}	Voltage-deactivation gate for I_{Ks}
r_{k1}	Voltage-rectifier gate for I_{K1}
aa	Voltage-activation gate for I_{to2}
r_{ito2}	Voltage-rectifier gate for I_{to2}

k_{ito2}	Ca^{+}-activation gate for I_{to2}
x_{kb}	Voltage-rectifier gate for I_{Kb}
τ_x	Time constant of gate x (ms)

Conductances and Permeability

G_{Na}	Maximum conductance of I_{Na} (mS/µF)
G_{NaL}	Maximum conductance of I_{NaL} (mS/µF)
P_{Ca}	Membrane permeability to Ca^{2+} in L-type channels (cm/s)
P_{CaNa}	Membrane permeability to Na^{+} in L-type channels (cm/s)
P_{CaK}	Membrane permeability to K^{+} in L-type channels (cm/s)
G_{Kr}	Maximum conductance of I_{Kr} (mS/µF)
G_{Ks}	Maximum conductance of I_{Ks} (mS/µF)
G_{K1}	Maximum conductance of I_{K1} (mS/µF)
G_{to2}	Maximum conductance of I_{to2} (mS/µF)
G_{Kb}	Maximum conductance of I_{Kb} (mS/µF)
P_{Nab}	Membrane permeability of Na^{+} in I_{Nab} (cm/s)
P_{Cab}	Membrane permeability of Ca^{2+} in I_{Cab} (cm/s)
G_{pCa}	Maximum conductance of I_{pCa} (mS/µF)
G_{relol}	Ca^{2+}-overload induced SR Ca^{2+} release strength (1/ms)

Concentrations

Na_o	Extracellular Na^{+} concentration (mM)
Na_i	Intracellular Na^{+} concentration in the myoplasm (mM)
Na_{ss}	Intracellular Na^{+} concentration in the subspace (mM)
K_o	Extracellular K^{+} concentration (mM)
K_i	Intracellular K^{+} concentration in the myoplasm (mM)
K_{ss}	Intracellular K^{+} concentration in the subspace (mM)
Cl_i	Intracellular Cl^{-} concentration in the myoplasm (mM)
Cl_o	Extracellular Cl^{-} concentration (mM)

Ca_i	Average Ca^{2+} concentration in the myoplasm (µM)
Ca_{i1}	Ca^{2+} concentration in the t-tubular myoplasm (µM)
Ca_{i2}	Ca^{2+} concentration in the non t-tubular myoplasm (µM)
Ca_{ss}	Ca^{2+} concentration in the subspace (mM)
Ca_{JSR}	Ca^{2+} concentration in the JSR (mM)
Ca_{CSR}	Ca^{2+} concentration in the CSR (mM)
$Ca_{JSRthol}$	Threshold for Ca^{2+} concentration in JSR for Ca^{2+} overload induced spontaneous SR Ca^{2+} release (mM)
Ca_{NSR}	Ca^{2+} concentration in the NSR (mM)

Fluxes

J_{rel1}	SR Ca^{2+} release in the subspace (mM/ms)
J_{gap}	Ca^{2+} diffusion between the t-tubular and the non t-tubular region (mM/ms)
J_{rel2}	SR Ca^{2+} release in the non t-tubular region (mM/ms)
J_{relol}	Ca^{2+}-overload induced spontaneous SR Ca^{2+} release (mM/ms)
J_{leak}	Ca^{2+} leak through the NSR (mM/ms)
J_{upnp1}	SR Ca^{2+} uptake via non-phosphorylated SERCA pump in the t-tubular region (mM/ms)
J_{upp1}	SR Ca^{2+} uptake via CaMK phosphorylated SERCA pump in the t-tubular region (mM/ms)
J_{up1}	Total SR Ca^{2+} uptake via SERCA pump in the t-tubular region (mM/ms)
J_{upnp2}	SR Ca^{2+} uptake via non-phosphorylated SERCA pump in the non t-tubular region (mM/ms)
J_{upp2}	SR Ca^{2+} uptake via CaMK phosphorylated SERCA pump in the non t-tubular region (mM/ms)
J_{up2}	Total SR Ca^{2+} uptake via SERCA pump in the non t-tubular region (mM/ms)
J_{tr1}	Ca^{2+} translocation from the NSR to the JSR (mM/ms)

J_{tr2}	Ca^{2+} translocation from the NSR to the CSR (mM/ms)

Buffers

CMDN	Calmodulin Ca^{2+} buffer in the myoplasm
TRPN	Troponin Ca^{2+} buffer in the myoplasm
BSR	Anionic SR binding sites for Ca^{2+} in the subspace
BSL	Anionic sarcolemmal binding sites for Ca^{2+} in the subspace
CSQN	Calsequestrin Ca^{2+} buffer in the JSR
β_Y	Buffer factor for compartment Y

Reversal Potentials

E_{Na}	Reversal potential for I_{Na} and I_{NaL} (mV)
E_K	Reversal potential for I_{Kr} and I_{K1} (mV)
E_{Ks}	Reversal potential for I_{Ks} (mV)
E_{Cl}	Reversal potential for I_{to2} (mV)

Others

C_m	Cellular membrane capacitance ($\mu F/cm^2$)
V_m	Membrane potential (mV)
F	Faraday constant (C/mol)
R	Gas constant (J/kmol/K)
T	Temperature (K)
CL	Cycle Length (ms)
t_{ol}	Time of start of Ca^{2+} overload-induced spontaneous SR Ca^{2+} release (ms)
z_{Ca}	Valence state of Ca^{2+}
z_{Na}	Valence state of Na^{+}
z_K	Valence state of K^{+}
$\gamma_{Nai}, \gamma_{Nao}$	Activity coefficient of Na^{+}
$\gamma_{Cai}, \gamma_{Cao}$	Activity coefficient of Ca^{2+}

$PR_{Na,K}$	Permeability ratio of Na^+ to K^+
CaMK	Ca^{2+}/Calmodulin-dependent protein kinase
$CaMK_{trap}$	Trapped CaMK concentration (mM)
$CaMK_{bound}$	Bound CaMK concentration (mM)
$CaMK_{active}$	Active CaMK concentration (mM)
SERCA	Sarcoendoplasmic reticulum Ca^{2+} ATPase
PLB	Phospholamban

MODEL PARAMETERS

Cell Geometry

$$L_{cell} = 0.017$$

$$R_{cell} = 0.0011$$

$$V_{cell} = 1000 \times \pi \times R_{cell}^2 \times L_{cell}$$

$$A_{geo} = 2 \times \pi \times R_{cell}^2 + 2 \times \pi \times R_{cell} \times L_{cell}$$

$$A_{cap} = 2 \times A_{geo}$$

$$V_{myo} = 0.68 \times V_{cell}$$

$$V_{myo1} = 0.34 \times V_{cell}$$

$$V_{myo2} = V_{myo} - V_{myo1}$$

$$V_{NSR} = 0.0552 \times V_{cell}$$

$$V_{NSR1} = 0.0276 \times V_{cell}$$

$$V_{NSR2} = 0.0276 \times V_{cell}$$

$$V_{JSR} = 0.0024 \times V_{cell}$$

$$V_{CSR} = 0.0024 \times V_{cell}$$

$$V_{ss} = 0.01 \times V_{cell}$$

The values for t-tubular signal density in pig myocytes were ~50% of the values obtained for mouse myocytes [1]. Mouse myocytes have homogeneously distributed t-tubular network. Accordingly, we set the t-tubular and the non t-tubular region of pig myocyte as 50% of total pig myocyte volume. JSR is in t-tubular region and CSR is in non t-tubular region. Subspace is in t-

tubular region. Sources of parameter values are mentioned in the table MODEL parameters and formulation sources at the end of this Data Supplement.

Fixed Ionic Concentrations

$$\mathrm{Na_o} = 140$$

$$\mathrm{K_o} = 5.4$$

$$\mathrm{Ca_o} = 5.4$$

$$\mathrm{Cl_o} = 100$$

$$\mathrm{Cl_i} = 19.53$$

Initial Conditions (at CL=1000 ms)

$$\mathrm{V_m} = -87.3$$

$$\mathrm{Na_i} = 6.43$$

$$\mathrm{Na_{ss}} = 6.43$$

$$\mathrm{K_i} = 140.65$$

$$\mathrm{K_{ss}} = 140.65$$

$$\mathrm{Ca_{i1}} = 7.054 \times 10^{-5}$$

$$\mathrm{Ca_{i2}} = 7.135 \times 10^{-5}$$

$$\mathrm{Ca_{ss}} = 7.015 \times 10^{-5}$$

$$\mathrm{Ca_{JSR}} = 1.18$$

$$\mathrm{Ca_{NSR}} = 1.25$$

$$\mathrm{Ca_{CSR}} = 1.19$$

$$\mathrm{CaMK_{trap}} = 0.00814$$

$$\mathrm{J_{rel1}} = 0$$

$$\mathrm{J_{rel2}} = 0$$

$$\mathrm{aa} = 1$$

$$\mathrm{d} = 0$$

$$\mathrm{f_{Ca}} = 0.99$$

$$\mathrm{ff} = 1$$

$$fs = 1$$

$$h = 0.63$$

$$hl = 0.3$$

$$j = 0.63$$

$$m = 0$$

$$ml = 0$$

$$xr = 0.153$$

$$xs1 = 0.054$$

$$xs2 = 0.046$$

Physical Constants

$$R = 8314$$

$$T = 310$$

$$F = 96485$$

Reversal Potentials

$$E_{Na} = \left(\frac{R \times F}{T}\right) \times \log\left(\frac{Na_o}{Na_i}\right)$$

$$E_K = \left(\frac{R \times F}{T}\right) \times \log\left(\frac{K_o}{K_i}\right)$$

$$E_{Cl} = \left(\frac{R \times F}{T}\right) \times \log\left(\frac{Cl_i}{Cl_o}\right)$$

$$PR_{Na,K} = 0.01833$$

$$E_{Ks} = \left(\frac{R \times F}{T}\right) \times \log\left(\frac{K_o + PR_{Na,K} \times Na_o}{K_i + PR_{Na,K} \times Na_i}\right)$$

MODEL ELECTROPHYSIOLOGY

Fast Sodium Current (I_{Na})

$$G_{Na} = 25$$

$$m_{ss} = \frac{1}{1 + \exp\left(\frac{49.4 - V_m}{6.2}\right)}$$

$$\tau_m = \frac{0.25}{7 \times \exp\left(\frac{V_m + 10}{35}\right) + 10 \times \exp\left(\frac{-80 - V_m}{6}\right)}$$

$$h_{ss} = \frac{1}{1 + \exp\left(\frac{84.2 + V_m}{5.8}\right)}$$

$$\tau_h = \frac{1}{10^{-5} \times \exp\left(\frac{-V_m - 1}{6}\right) + 6 \times \exp\left(\frac{0.5 + V_m}{20}\right)}$$

$$j_{ss} = h_{ss}$$

$$\tau_j = 2 + \frac{1}{0.02 \times \exp\left(\frac{-V_m - 100}{8}\right) + 0.3 \times \exp\left(\frac{1 + V_m}{40}\right)}$$

$$I_{Na} = G_{Na} \times m^3 \times h \times j \times (V_m - E_{Na})$$

Late Sodium Current (I_{NaL})

$$G_{NaL} = 0.0075$$

$$a_{ml} = \frac{0.32 \times (V_m + 47.13)}{1 - \exp(-0.1 \times (V_m + 47.13))}$$

$$b_{ml} = 0.08 \times \exp\left(\frac{-V_m}{11}\right)$$

$$ml_{ss} = \frac{a_{ml}}{a_{ml} + b_{ml}}$$

$$\tau_{ml} = \frac{1}{a_{ml} + b_{ml}}$$

$$hl_{ss} = \frac{1}{1 + \exp\left(\frac{V_m + 91}{6.1}\right)}$$

$$\tau_{hl} = 600$$

$$I_{NaL} = G_{NaL} \times ml^3 \times hl \times (V_m - E_{Na})$$

L-type Current

$$d_{ss} = \frac{1}{1 + \exp\left(\frac{-V_m + 3.4}{6.2}\right)}$$

$$\tau_d = 0.6 + \frac{1}{\exp\left(-0.05 \times (V_m + 6)\right) + \exp\left(0.09 \times (V_m + 14)\right)}$$

$$f_{ss} = \frac{1}{1 + \exp\left(\frac{V_m + 22.9}{4.9}\right)} + \frac{0.35}{1 + \exp\left(\frac{-V_m + 45}{20}\right)}$$

$$ff_{ss} = f_{ss}$$

$$fs_{ss} = f_{ss}$$

$$f = ff \times fs$$

$$\tau_{ff} = 7 + \frac{1}{0.0045 \times \exp\left(\frac{-(V_m + 20)}{10}\right) + 0.0045 \times \exp\left(\frac{V_m + 20}{10}\right)}$$

$$\tau_{fs} = 70 + \frac{1}{3.5 \times 10^{-5} \times \exp\left(\frac{-(V_m + 5)}{4}\right) + 3.5 \times 10^{-5} \exp\left(\frac{V_m + 5}{6}\right)}$$

$$f_{Cass} = \frac{0.3}{1 - \frac{I_{CaL}}{0.05}} + \frac{0.55}{1 + \frac{Ca_{ss}}{0.003}} + 0.15$$

$$\tau_{fCa} = 10 \times \frac{CaMK_{active}}{K_{m,CaMK} + CaMK_{active}} + 0.5 + \frac{1}{1 + \frac{Ca_{ss}}{0.003}}$$

$$P_{Ca} = 2 \times 10^{-4}$$

$$\gamma_{Cai} = 1.0; \gamma_{Cao} = 0.341; z_{Ca} = 2$$

$$\phi_{Ca} = z_{Ca}^2 \times \frac{V_m \times F^2}{R \times T} \times \frac{\gamma_{Cai} \times Ca_{ss} \times \exp\left(\frac{z_{Ca} \times V_m \times F}{R \times T}\right) - \gamma_{Cao} \times Ca_o}{\exp\left(\frac{z_{Ca} \times V_m \times F}{R \times T}\right) - 1.0}$$

$$P_{CaNa} = 0.00125 \times P_{Ca}; \gamma_{Nai} = 0.75; \gamma_{Nao} = 0.75; z_{Na} = 1$$

$$\phi_{CaNa} = z_{Na}^2 \times \frac{V_m \times F^2}{R \times T} \times \frac{\gamma_{Nai} \times Na_{ss} \times \exp\left(\frac{z_{Na} \times V_m \times F}{R \times T}\right) - \gamma_{Nao} \times Na_o}{\exp\left(\frac{z_{Na} \times V_m \times F}{R \times T}\right) - 1.0}$$

$$P_{CaK} = 3.574 \times 10^{-4} \times P_{Ca}; \gamma_{Ki} = 0.75; \gamma_{Ko} = 0.75; z_K = 1$$

$$\phi_{CaK} = z_K^2 \times \frac{V_m \times F^2}{R \times T} \times \frac{\gamma_{Ki} \times K_{ss} \times \exp\left(\frac{z_K \times V_m \times F}{R \times T}\right) - \gamma_{Ko} \times K_o}{\exp\left(\frac{z_K \times V_m \times F}{R \times T}\right) - 1.0}$$

$$I_{CaL} = \phi_{Ca} \times d \times f \times f_{Ca}$$

$$I_{CaNa} = \phi_{CaNa} \times d \times f \times f_{Ca}$$

$$I_{CaK} = \phi_{CaK} \times d \times f \times f_{Ca}$$

Rapid component of delayed rectifier K+ current (I_{Kr})

$$G_{Kr} = 0.0075;$$

$$x_{rss} = \frac{1}{1 + \exp\left(\frac{-(V_m + 56.8)}{17.8}\right)}$$

$$\tau_{xr} = 12.98 + \frac{1}{0.3652 \times \exp\left(\frac{V_m - 31.66}{3.869}\right) + 4.123 \times 10^{-5} \exp\left(\frac{-(V_m - 47.78)}{20.38}\right)}$$

$$r_{Kr} = \frac{1}{1 + \exp\left(\frac{V_m + 22}{15}\right)}$$

$$I_{Kr} = G_{Kr} \times \sqrt{\frac{K_o}{5.4}} \times x_r \times r_{Kr} \times (V_m - E_K)$$

Slow component of delayed rectifier K+ current (I_{Ks})

$$G_{Ks} = 0.021$$

$$x_{s1ss} = \frac{1}{1 + \exp\left(\frac{-(V_m - 25.1)}{37.1}\right)}$$

$$\tau_{xs1} = 1750 + \frac{1}{2.326^{-6} \times \exp\left(\frac{V_m + 28.28}{17.8}\right) + 0.001292 \times \exp\left(\frac{-(V_m + 210)}{230}\right)}$$

$$x_{s2ss} = x_{s1ss}$$

$$\tau_{xs2} = \frac{1}{0.01 \times \exp\left(\frac{V_m - 50.0}{20.0}\right) + 0.0193 \times \exp\left(\frac{-(V_m + 66.54)}{31.0}\right)}$$

$$K_{sCa} = 1 + \frac{0.6}{1 + \left(\frac{3.8 \times 10^{-5}}{Ca_{i1}}\right)^{1.4}}$$

$$I_{Ks} = G_{Ks} \times K_{sCa} \times x_{s1} \times x_{s2} \times (V_m - E_{Ks})$$

Inward rectifier K+ current (I_{K1})

$$G_{K1} = 0.21$$

$$r_{K1} = \frac{1}{1 + \exp\left(\frac{V_m + 79.3 - 2.6 \times K_o}{19.6}\right)}$$

$$I_{K1} = G_{K1} \times \sqrt{\frac{K_o}{5.4}} \times r_{K1} \times (V_m - E_K)$$

Ca^{2+}-activated Cl^- current (I_{to2})

$$G_{to2} = 0.2$$

$$k_{to2} = 1 - \frac{1}{1 + \left(\frac{J_{rel1}}{0.4}\right)^2}$$

$$\alpha_{aa} = \frac{0.025}{1 + \exp\left(\frac{V_m + 58}{5}\right)}$$

$$\beta_{aa} = \frac{1}{5 \times \left(1 + \exp\left(\frac{-(V_m + 19)}{9}\right)\right)}$$

$$aa_{ss} = \frac{\alpha_{aa}}{\alpha_{aa} + \beta_{aa}}$$

$$\tau_{aa} = \frac{1}{\alpha_{aa} + \beta_{aa}}$$

$$r_{to2} = \frac{1}{1 + \exp\left(\frac{-(V_m + 10)}{5}\right)}$$

$$I_{to2} = G_{to2} \times aa \times r_{to2} \times k_{to2} \times (V_m - E_{Cl})$$

Na^+-Ca^{2+} exchanger current (I_{NaCa})

$$G_{NaCa} = 0.0008$$

The mathematical equations describing I_{NaCa} are the same as ORd [2]. I_{NaCa} is a function of V_m and intracellular and extracellular ionic concentrations of Ca^{2+} and Na^+. 80% of Na^+-Ca^{2+} exchanger protein is assumed to be located in the myoplasm and 20% in the subspace [2].

Na^+- K^+ ATPase current (I_{NaK})

$$P_{NaK} = 30$$

The mathematical equations of I_{NaK} are same as ORd [2]. I_{NaK} is critically dependent on V_m and intracellular/extracellular concentrations of sodium ion (Na^+) and potassium ion (K^+). It maintains intracellular homeostasis of Na^+ and K^+ [2].

Background K^+ current (I_{Kb})

$$G_{Kb} = 0.003$$

$$x_{Kb} = \frac{1}{1 + \exp\left(\frac{-(V_m - 14.48)}{4.34}\right)}$$

$$I_{Kb} = G_{Kb} \times x_{Kb} \times (V_m - E_K)$$

Background Na^+ current (I_{Nab})

$$P_{Nab} = 3.75 \times 10^{-10}, z_{Na} = 1$$

$$I_{Nab} = P_{Nab} \times z_{Na}^2 \times \left(\frac{V_m \times F \times F}{R \times T}\right) \times \left(\frac{Na_i \times \exp\left(\frac{V_m \times F}{R \times T}\right) - Na_o}{\exp\left(\frac{V_m \times F}{R \times T}\right) - 1.0}\right)$$

Background Ca^{2+} current (I_{Cab})

$$P_{Cab} = 2.5 \times 10^{-8}, z_{Ca} = 2, \gamma_{Cai} = 1.0, \gamma_{Cao} = 0.341$$

$$I_{Cab} = P_{Cab} \times z_{Ca}^2 \times \left(\frac{V_m \times F \times F}{R \times T}\right) \times \left(\frac{\gamma_{Cai} \times Ca_{i1} \times \exp\left(\frac{V_m \times F}{R \times T}\right) - \gamma_{Cao} \times Ca_o}{\exp\left(\frac{V_m \times F}{R \times T}\right) - 1.0}\right)$$

Sarcolemmal Ca^{2+} pump current (I_{pCa})

$$G_{pCa} = 0.0575$$

$$I_{pCa} = G_{pCa} \times \left(\frac{Ca_{i1}}{0.0005 + Ca_{i1}}\right)$$

MODEL Membrane voltage (V_m)

$$\frac{dV_m}{dt} = -(I_{Na} + I_{NaL} + I_{CaL} + I_{CaNa} + I_{CaK} + I_{Kr} + I_{Ks} + I_{K1} + I_{To2} + I_{NaCai} + I_{NaCass} + I_{NaK} + I_{Nab} + I_{Kb} + I_{pCa} + I_{Cab})$$

MODEL Ca^{2+} handling

Ca^{2+}/Calmodulin-dependent Protein Kinase (CaMK)

$$\alpha_{CaMK} = 0.05$$

$$\beta_{CaMK} = 0.00068$$

$$CaMK_0 = 0.05$$

$$K_{mCaM} = 0.0015$$

$$K_{mCaMK} = 0.065$$

$$CaMK_{bound} = CaMK_0 \times \frac{1 - CaMK_{trap}}{1 + \frac{K_{mCaM}}{Ca_{ss}}}$$

$$CaMK_{active} = CaMK_{bound} + CaMK_{trap}$$

$$\phi_{CaMK} = \frac{1}{1 + \frac{K_{mCaMK}}{CaMK_{active}}}$$

$$\frac{dCaMK_{trap}}{dt} = \alpha_{CaMK} \times CaMK_{bound} \times CaMK_{active} - \beta_{CaMK} \times CaMK_{trap}$$

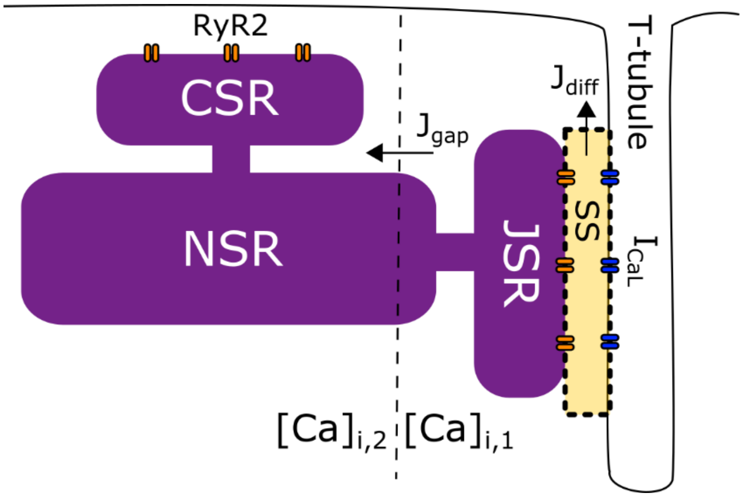

Figure A: Schematic of 2-step calcium release. Ryanodine (RyR2) receptors are shown in orange with L-type calcium channels shown in blue (I_{CaL}). The corbular sarcoplasmic reticulum sees calcium concentration in non t-tubular space, $[Ca]_{i,2}$. Calcium diffuses from the subspace (yellowish region labelled SS) to the intracellular space near the t-tubule (associated calcium concentration $[Ca]_{i,1}$) with flux J_{diff}. Calcium flux between the intracellular t-tubular and non t-tubular spaces, J_{gap}, occurs with a time constant of τ_{gap}.

Myoplasmic Ca^{2+} Buffers

$$CMDN_{max} = 0.05; K_{mCMDN} = 0.00238$$

$$TRPN_{max} = 0.07; K_{mTRPN} = 0.0005$$

Subspace Ca^{2+} Buffers

$$BSR_{max} = 0.047; K_{mBSR} = 0.00087$$

$$BSL_{max} = 1.124; K_{mBSL} = 0.0087$$

Sarcoplasmic Reticulum (SR) Buffer

$$\mathrm{CSQN_{max}} = 10.0, \mathrm{K_{mCSQN}} = 0.8$$

SR Ca^{2+} SERCA/PLB Uptake flux

$$\mathrm{J_{upnp1}} = 0.005 \times \frac{\mathrm{Ca_{i1}}}{\mathrm{Ca_{i1}} + 0.001}$$

$$\mathrm{J_{upp1}} = 0.015 \times \frac{\mathrm{Ca_{i1}}}{\mathrm{Ca_{i1}} + 0.0008}$$

$$\mathrm{J_{leak}} = 0.005 \times \frac{\mathrm{Ca_{NSR}}}{15}$$

$$\mathrm{J_{up1}} = (1.0 - \phi_{\mathrm{CaMK}}) \times \mathrm{J_{upnp1}} + \phi_{\mathrm{CaMK}} \times \mathrm{J_{upp1}} - \mathrm{J_{leak}}$$

$$\mathrm{J_{upnp2}} = 0.005 \times \frac{\mathrm{Ca_{i2}}}{\mathrm{Ca_{i2}} + 0.001}$$

$$\mathrm{J_{upp2}} = 0.015 \times \frac{\mathrm{Ca_{i2}}}{\mathrm{Ca_{i2}} + 0.0008}$$

$$\mathrm{J_{up2}} = (1.0 - \phi_{\mathrm{CaMK}}) \times \mathrm{J_{upnp2}} + \phi_{\mathrm{CaMK}} \times \mathrm{J_{upp2}} - \mathrm{J_{leak}}$$

Ca^{2+} translocation flux between junctional/corbular SR and network SR

$$\mathrm{J_{tr1}} = \frac{\mathrm{Ca_{NSR}} - \mathrm{Ca_{JSR}}}{100}$$

$$\mathrm{J_{tr2}} = \frac{\mathrm{Ca_{NSR}} - \mathrm{Ca_{CSR}}}{100}$$

Diffusion flux from subspace to myoplasm in t-tubular region

$$\mathrm{J_{diff}} = \frac{\mathrm{Ca_{ss}} - \mathrm{Ca_{i1}}}{0.2}$$

SR Ca^{2+} release from subspace in t-tubular region

$$\mathrm{Rel1} = (-\mathrm{I_{CaL}} + 2 \times \mathrm{I_{NaCass}}) \times \left(\frac{\mathrm{C_m} \times \mathrm{A_{cap}}}{2 \times \mathrm{V_{ss}} \times \mathrm{F}}\right) + \mathrm{J_{rel1}} \times \left(\frac{\mathrm{V_{JSR}}}{\mathrm{V_{ss}}}\right) - \mathrm{J_{diff}}$$

$$\tau_{Jrel1} = 20 \times \frac{\left(1 + \frac{1}{1 + \left(\frac{K_{mCaMK}}{CaMK_{active}}\right)^8}\right)}{1 + \left(\frac{0.5}{Ca_{JSR}}\right)^8}$$

$$\text{if } \tau_{Jrel1} < 0.001, \tau_{Jrel1} = 0.001$$

$$\text{if Rel1} > 0$$

$$J_{rel1ss} = 60 \times Rel1 \times \frac{\left(1 + \frac{1}{1 + \left(\frac{K_{mCaMK}}{CaMK_{active}}\right)^8}\right)}{1 + \left(\frac{0.75}{Ca_{JSR}}\right)^8}$$

$$\text{else } J_{rel1ss} = 0$$

SR Ca^{2+} release in the non t-tubular region

$$Rel2 = J_{gap} + J_{rel2} \times \left(\frac{V_{CSR}}{V_{myo2}}\right) - J_{up2} \times \left(\frac{V_{NSR2}}{V_{myo2}}\right)$$

$$\tau_{Jrel2} = 50 \times \frac{\left(1 + \frac{1}{1 + \left(\frac{K_{mCaMK}}{CaMK_{active}}\right)^8}\right)}{1 + \left(\frac{0.5}{Ca_{CSR}}\right)^8}$$

$$\text{if } \tau_{Jrel2} < 0.001, \tau_{Jrel2} = 0.001$$

$$\text{if Rel2} > 0$$

$$J_{rel2ss} = 250 \times Rel2 \times \frac{\left(1 + \frac{1}{1 + \left(\frac{K_{mCaMK}}{CaMK_{active}}\right)^8}\right)}{1 + \left(\frac{0.75}{Ca_{CSR}}\right)^8}$$

$$\text{else } J_{rel1ss} = 0$$

Ca^{2+} diffusion flux between the t-tubular and the non t-tubular region

$$\tau_{gap} = 3$$

$$J_{gap} = \frac{Ca_{i1} - Ca_{i2}}{\tau_{gap}}$$

Spontaneous Overload Induced SR Ca^{2+} release (SOICR) by Ryanodine Receptors (J_{relol})

When the SR Ca^{2+} levels reaches a certain level, spontaneous SR Ca^{2+} release occurs in the cardiac myocytes in the absence of membrane depolarization [3,4]. This depolarization-independent Ca^{2+}-overload induced spontaneous release (termed SOICR [5]) formulation is modified from the Luo-Rudy model [6]. It is assumed that the spontaneous SR Ca^{2+} release occurs from the JSR [7].

$$\tau_{olon} = 0.5$$

$$\tau_{oloff} = 5$$

$$G_{relol}^{max} = 2$$

$$G_{relol} = G_{relol}^{max} \times \left(1 - \exp\left(\frac{-t_{ol}}{\tau_{olon}}\right)\right) \times \exp\left(\frac{-t_{ol}}{\tau_{oloff}}\right)$$

$$J_{relol} = G_{relol} \times \left(Ca_{JSR} - Ca_{ss}\right)$$

Ca^{2+} concentrations in myoplasm, subspace, network SR, junctional SR and corbular SR

$$\beta_{Cai1} = \frac{1}{1 + \frac{CMDN_{max} \times K_{mCMDN}}{(K_{mCMDN} + Ca_{i1})^2} + \frac{TRPN_{max} \times K_{mTRPN}}{(K_{mTRPN} + Ca_{i1})^2}}$$

$$\frac{dCa_{i1}}{dt} = \beta_{Cai1} \times \left(-\left(I_{pCa} + I_{Cab} - 2.0 \times I_{NaCai}\right) \times \left(\frac{C_m \times A_{cap}}{2 \times F \times V_{myo1}}\right) - J_{up1} \times \left(\frac{V_{NSR1}}{V_{myo1}}\right) + J_{diff} \times \left(\frac{V_{ss}}{V_{myo1}}\right) - J_{gap}\right)$$

$$\beta_{Cai2} = \frac{1}{1 + \frac{CMDN_{max} \times K_{mCMDN}}{(K_{mCMDN} + Ca_{i2})^2} + \frac{TRPN_{max} \times K_{mTRPN}}{(K_{mTRPN} + Ca_{i2})^2}}$$

$$\frac{dCa_{i2}}{dt} = \beta_{Cai2} \times \left(J_{rel2} \times \left(\frac{V_{CSR}}{V_{myo2}}\right) + J_{gap} \times \left(\frac{V_{myo1}}{V_{myo2}}\right) - J_{up2} \times \left(\frac{V_{NSR2}}{V_{myo2}}\right)\right)$$

$$Ca_i = \left(\frac{V_{myo1}}{V_{myo}}\right) \times Ca_{i1} + \left(\frac{V_{myo2}}{V_{myo}}\right) \times Ca_{i2}$$

$$\beta_{Cass} = \frac{1}{1 + \frac{BSR_{max} \times K_{mBSR}}{(K_{mBSR} + Ca_{ss})^2} + \frac{BSL_{max} \times K_{mBSL}}{(K_{mBSL} + Ca_{ss})^2}}$$

$$\frac{dCa_{ss}}{dt} = \beta_{Cass} \times \left(-(I_{CaL} - 2.0 \times I_{NaCass}) \times \left(\frac{C_m \times A_{cap}}{2 \times F \times V_{ss}}\right) + (J_{rel1} + J_{relol}) \times \left(\frac{V_{JSR}}{V_{ss}}\right) - J_{diff}\right)$$

$$\beta_{CaJSR} = \frac{1}{1 + \frac{CSQN_{max} \times K_{mCSQN}}{\left(K_{mCSQN} + Ca_{JSR}\right)^2}}$$

$$\frac{dCa_{JSR}}{dt} = \beta_{CaJSR} \times (J_{tr1} - J_{rel1} - J_{relol})$$

$$\beta_{CaCSR} = \frac{1}{1 + \frac{CSQN_{max} \times K_{mCSQN}}{\left(K_{mCSQN} + Ca_{CSR}\right)^2}}$$

$$\frac{dCa_{CSR}}{dt} = \beta_{CaCSR} \times (J_{tr2} - J_{rel2})$$

$$\frac{dCa_{NSR}}{dt} = J_{up1} \times \left(\frac{V_{NSR1}}{V_{NSR}}\right) + J_{up2} \times \left(\frac{V_{NSR2}}{V_{NSR}}\right) - J_{tr1} \times \left(\frac{V_{JSR}}{V_{NSR}}\right) - J_{tr2} \times \left(\frac{V_{CSR}}{V_{NSR}}\right)$$

MODEL Na^+ handling

Na^+ diffusion flux from subspace to myoplasm

$$J_{diffNa} = \frac{Na_{ss} - Na_i}{2}$$

Na^+ concentration in the subspace and myoplasm

$$\frac{dNa_{ss}}{dt} = -(I_{CaNa} + 3 \times I_{NaCass}) \times \left(\frac{C_m \times A_{cap}}{F \times V_{ss}}\right) - J_{diffNa}$$

$$\frac{dNa_i}{dt} = -(I_{Na} + I_{NaL} + 3 \times I_{NaCai} + 3 \times I_{NaK} + I_{Nab}) \times \left(\frac{C_m \times A_{cap}}{F \times V_{ss}}\right) + J_{diffNa} \times \left(\frac{V_{ss}}{V_{myo}}\right)$$

MODEL K^+ handling

K^+ diffusion flux from subspace to myoplasm

$$J_{diffK} = \frac{K_{ss} - K_i}{2}$$

K^+ concentrations in the subspace and myoplasm

$$\frac{dK_{ss}}{dt} = -I_{CaK} \times \left(\frac{A_{cap}}{F \times V_{ss}}\right) - J_{diffK}$$

$$\frac{dK_i}{dt} = -(I_{Kr} + I_{Ks} + I_{K1} + I_{Kb} - 2 \times I_{NaK}) \times \left(\frac{A_{cap}}{F \times V_{myo}}\right) + J_{diffK} \times \left(\frac{V_{ss}}{V_{myo}}\right)$$

Table A: MODEL parameters and formulation sources. Gold shaded parameters used experimental data for validation.

Parameter	Source	Parameter	Source
**Cell Geometry**			
L_{cell}	[8]	V_{NSR1}	[1,9]
R_{cell}	[9]	V_{NSR2}	[1,9]
A_{cap}	[9]	V_{JSR}	[1,9]
V_{myo}, V_{NSR}	[9]	V_{CSR}	[1,9]
V_{myo1}	[1,9]	V_{ss}	[1,9]
V_{myo2}	[1,9]		
**MODEL Electrophysiology**			
I_{Na}		**L-type current**	
G_{Na}	Match dV/dt from [8] (Fig 2B)	d_{ss}	Expt (Fig 1C)
m_{ss}	[8] (Fig 1A)	τ_d	[2]
τ_m	Modified from [2] to match dV/dt (Fig 2B)	f_{ss}	Expt (Fig 1B)
h_{ss}	[2]	τ_{ff}	[2]
τ_h	[2]	τ_{fs}	Modified from [2]
τ_j	[2]	f_{Cass}	$f_{Ca,\infty}$ in [10]
		τ_{fCa}	τ_{fCa} in [10]
I_{NaL}		P_{Ca}	APD match (Fig 2E)
G_{NaL}	[10]	I_{Kr}	
a_{ml}, b_{ml}, ml_{ss}, τ_{hl}	[10]	G_{Kr}	APD match (Fig 2E)
I_{K1}		x_{rss}	Expt I_{Kr} I-V in [8] (Fig 1E)
G_{K1}	APD (Fig 2E)	τ_{xr}	$\tau_{xr,fast}$ in [2]
r_{K1}	I_{K1} I-V curve (Fig 1H)	r_{Kr}	Expt I_{Kr} I-V in [8] (Fig 1E)

I_{K1} formulation	Modified from [2]	I_{Kr} formulation	[10]
**I_{to2}**		**I_{Kb}**	
**G_{To2}**	AP notch shape (Fig 2B)	G_{Kb}	[2]
**k_{To2}**	[11]	x_{Kb}	[2]
**α_{aa}**	[11]	I_{Kb} formulation	[2]
**β_{aa}**	[11]		
**r_{To2}**	AP shape (Fig 2B)	I_{Nab} parameters and formulation	[2]
**I_{Ks}**		I_{NaCa} parameters and formulation	[2]
G_{Ks}, $xs1_{ss}$	I_{Ks} I-V curve [8] (Fig 1F)	I_{NaK} parameters and formulation	[2]
**τ_{xs1}**	Expt activation time constant (Fig 1G)	I_{Cab} parameters and formulation	[2]
**τ_{xs2}**	[2]	I_{pCa} parameters and formulation	[5]
I_{Ks} formulation	[2]		
**MODEL Ca^{2+} handling**			
CaMK parameters and formulation	[10]	Ca^{2+} translocation from NSR to JSR and CSR	
Ca^{2+} buffers parameters and formulations	[11]	J_{tr1}	[2]
SR Ca^{2+} SERCA/PLB Uptake flux parameters and formulations	Modified from [2]	J_{tr2}	[2]
**τ_{gap}**	Expt Ca^{2+} transient morphology (Fig 2D)	Parameters and formulations of SR Ca^{2+} release in t-tubular and non t-tubular region	Modified from [12] to match Ca^{2+} transient morphology (Fig 2D)

Parameters and formulation of spontaneous overload induced SR Ca^{2+} release	Modified from [6]	J_{diff}	[2]
**MODEL Na^{+} and K^{+} handling**			
**J_{diffNa}**	[2]	J_{diffK}	[2]

References

1. Heinzel FR, Bito V, Volders PGA, Antoons G, Mubagwa K, Sipido KR. Spatial and temporal inhomogeneities during Ca2+ release from the sarcoplasmic reticulum in pig ventricular myocytes. Circ Res. 2002;91: 1023–1030. doi:10.1161/01.RES.0000045940.67060.DD

2. O'Hara T, Virág L, Varró A, Rudy Y. Simulation of the undiseased human cardiac ventricular action potential: Model formulation and experimental validation. PLoS Comput Biol. 2011;7. doi:10.1371/journal.pcbi.1002061

3. Kass RS, Tsien RW. Fluctuations in membrane current driven by intracellular calcium in cardiac Purkinje fibers. Biophys J. 1982;38: 259–269. doi:10.1016/S0006-3495(82)84557-8

4. Orchard CH, Eisner DA, Allen DG. Oscillations of intracellular Ca2+ in mammalian cardiac muscle. Nature. 1983;304: 735–738. doi:10.1038/304735a0

5. Jiang D, Xiao B, Yang D, Wang R, Choi P, Zhang L, et al. RyR2 mutations linked to ventricular tachycardia and sudden death reduce the threshold for store-overload-induced Ca2+ release (SOICR). Proc Natl Acad Sci U S A. 2004;101: 13062–13067. doi:10.1073/pnas.0402388101

6. Luo CH, Rudy Y. A dynamic model of the cardiac ventricular action potential: II. Afterdepolarizations, triggered activity, and potentiation. Circ Res. 1994;74: 1097–1113. doi:10.1161/01.RES.74.6.1097

7. Stern MD, Capogrossi MC, Lakatta EG. Spontaneous calcium release from the sarcoplasmic reticulum in myocardial cells: mechanisms and consequences. Cell Calcium. 1988;9: 247–256. doi:10.1016/0143-4160(88)90005-X

8. Verkerk AO, van Ginneken ACG, Berecki G, den Ruijter HM, Schumacher CA, Veldkamp MW, et al. Incorporated sarcolemmal fish oil fatty acids shorten pig ventricular action potentials. Cardiovasc Res. 2006;70: 509–520. doi:10.1016/j.cardiores.2006.02.022

9. Faber GM, Silva J, Livshitz L, Rudy Y. Kinetic properties of the cardiac L-type Ca2+ channel and its role in myocyte electrophysiology: A theoretical investigation. Biophys J. 2007;92: 1522–1543. doi:10.1529/biophysj.106.088807

10. Hund TJ, Rudy Y. Rate dependence and regulation of action potential and calcium

transient in a canine cardiac ventricular cell model. Circulation. 2004;110: 3168–74. doi:10.1161/01.CIR.0000147231.69595.D3

11. Decker KF, Heijman J, Silva JR, Hund TJ, Rudy Y. Properties and ionic mechanisms of action potential adaptation, restitution, and accommodation in canine epicardium. Am J Physiol - Hear Circ Physiol. 2009;296. doi:10.1152/ajpheart.01216.2008

12. Livshitz LM, Rudy Y. Regulation of Ca2+ and electrical alternans in cardiac myocytes: Role of CAMKII and repolarizing currents. Am J Physiol - Hear Circ Physiol. 2007;292. doi:10.1152/ajpheart.01347.2006
