# Supplementary material for: A computational model of pig ventricular cardiomyocyte electrophysiology and calcium handling: Translation from pig to human electrophysiology
Source: PLoS Comput Biol. 2021 Jun 30;17(6):e1009137. doi: 10.1371/journal.pcbi.1009137 (PMC8277015; doi:10.1371/journal.pcbi.1009137)
Supplement: Attachment — Submitted filename: Response.pdf [file pcbi.1009137.s003.pdf]

Reviewer's Responses to Questions

Comments to the Authors:
Please note here if the review is uploaded as an attachment.

Reviewer #1: The paper by Gaur et al. describes the development and implementation of a mathematical model of the pig ventricular myocyte, simulating the electrophysiology and calcium dynamics in the cell. The model is based on species-specific data, some of which were obtained as part of this study. Importantly, the authors also compare their pig myocyte model to an established model of the human ventricular myocyte, demonstrating important differences in the ionic currents and Ca dynamics that determine the action potential, its rate dependence, and susceptibility to arrhythmias. Given the importance of the pig (a large mammal) as an experimental animal in the study of cardiac arrhythmias and effects of drug, the study is timely and important. The data supplement contains the model equations, making the model available to researchers in the field.
Several issues should be addressed by the authors:
1. Figure 2C and 2D. While the model and experiment Ca traces are qualitatively similar in showing a fast rising phase followed by a slower phase, the actual morphology is very different. The rate dependence is also different, with the peak for 0.5Hz for example at 600ms in experiment and 200ms in the model. Can the authors improve the accuracy of simulating the experimental traces? If not, the differences between model and experiment should be discussed and their effects on the results and conclusions of the study should investigated and described.

Response: The authors acknowledge that the fit is not perfect. It shows the difficulty in fitting ionic models for both calcium and membrane voltage waveforms simultaneously over several frequencies, especially faster frequencies. To the authors' knowledge, this is not standard practice and attests to its difficulty, especially given the highly nonlinear nature of the problem. The ability to fit is also a limit of the mathematical models chosen to represent the underlying processes. The problem can be viewed as a mutli-objective optimization problem. We cannot exactly fill all requirements, and our optimal solution will depend on how we weigh the various fitting criteria, i.e., how we weigh the importance of fitting APD at 1 Hz versus diastolic [Ca] at 4 Hz. The number of possible parameters to alter is very large and some combination might produce a better fit, but we do not feel the additional considerable computation and time is justified, given the reasonableness of the current fit and the cellular variability observed, We have added a paragraph about this limitation in the Discussion section:

"Though the model recreates experimental behavior qualitatively, there are quantitative differences. While APD restitution was closely reproduced by the model, the changes in Ca transient shape did not occur at the same frequency, and the exact $[Ca^{2+}]_i$ was not known. However, major behavioral characteristics were captured within physiological bounds. This model does represent the best approximation of pig ventricular myocyte cellular electrophysiology and calcium handling to date."

Although the morphology of the Ca-transient differs between the experiment and the model in the timing of the second peak, the diastolic Ca-concentration and the duration of the Ca-transient is correctly represented in the model. These two parameters have been correlated with (patho)physiology (Baartscheer)(Sipido) and SR-function. Diastolic Ca-concentration is of primary importance for the genesis of delayed after depolarizations.(Baartscheer)

2. The Results section on alternans compares the simulations with biological experiments. What is missing in this section is comparison to the human model. You should add Panel C to Figure 8, showing simulations with the human model to demonstrate its greater sensitivity to alternans production. In the Discussion section on alternans, first paragraph, you mention APD alternans in the human model at CL=250ms and refer to Fig. 8, but Fig. 8 does not include these data.
Also, the last paragraph (Biological Experiments) is limited to Ca and does not mention the AP.

Response: The third panel C with the human model has been added, showing alternans. We show alternans of both the APD and the Ca-transient duration at CL 200 ms. AP is not mentioned in the last paragraph related to Fig. 8 because the detailed assessments performed with this protocol used only

intracellular Ca^{2+} as a biological measure. We did not simultaneously measure voltage and we did not repeat this extensive work in another series of studies measuring APs.

A paragraph on the human model has been added to the results.

3. The SR threshold for spontaneous Ca release was lower in the pig than in the human. Thus, pig does not require as much Ca accumulation in the SR (overload) for spontaneous release as human. Contrary to your statements, this alone will make the pig more susceptible to DADs and triggered activity. The fact that the pig is LESS vulnerable to these arrhythmic events is because IK1 is much larger in the pig than in the human, providing membrane stability and also the SR Ca load at fast rate is lower in the pig.

Response: The reviewer raises a good point. However, looking at levels alone is incomplete as there are mechanisms to regulate the calcium level that differ in strength between the 2 species. We have added this point to the discussion and have mentioned the role of the larger iK1 in relation to the DADs in the discussion (page 11):

"Although the absolute SOICR $[Ca]_i$ threshold was lower in the pig, which would suggest greater arrhythmogenic potential, porcine $[Ca]_{NSR}$ was lower and the pig had stronger compensatory mechanisms to protect against DADs."

4. Results, first paragraph, the sentence beginning with "E-4031 sensitive current…." Appears twice.

Response: Corrected

5. Rare dependence and restitution of APD. It says "….steady-state APD rate-dependence (restitution)…" --- it should be adaptation.

*Response: Restitution is a common term for this phenomenon. Clarified by adding "*indicating a greater rate-adaptation response".

6. Discussion, Ionic Differences …….. Please change to…. Differences between simulated and measured ionic currents.

Response: As requested

7. Discussion, Afterdepolarizations. The sentence " On the other hand…………as shown by the requirement for IK1 to produce DADs in pig." ……. It should be ...... by the requirement for 50% reduction in IK1 to produce DADs in pig.

Response: Done

8. Caption to Fig. 8. It will be useful to highlight (state) here that alternans were not produced in the pig, but were produced in human (after adding Panel C).

Response: Added as requested to the caption.

9. In Fig.8A, left, top – is the X axis time or CL - please indicate.

Response: This is now indicated in the caption as time.

10. In Fig. 8A, only the 1000ms traces sow the fast – slow phase. Please comment; why is the slow phase not present at the shorter CLs?

Response: Based on their model, Ca release has an initial rapid spike due to JSR release near the T-tubules and a second release from the CSR outside of this region, relying on diffusion from the first process. As pacing frequency increases, less Ca is released from the SR and hence, less Ca diffuses to the RyR receptors outside of the subspace. This triggers a much smaller and delayed secondary Ca release. [Ca] as imaged is an average of these two [Ca]'s. In the averaged response, the initial rise is JSR release which begins to decay rapidly. Whether there is a subsequent rise, plateau or decay depends on whether the rate of CSR release is larger, the same or less than the decay of JSR release. Thus, at rates faster than 1 Hz, CSR release is much weaker than the decay and only the fast response is observed, although the slow response is still contributes to prolonging the Ca transient observed. This is now explained in the text.

Reviewer #2: Review of "A Computational Model of Pig Ventricular Cardiomyocyte Electrophysiology and Calcium Handling: Translation from Pig to Human Electrophysiology"
By Namit Guar, Xiao-Yan Qi, David Benoist, Olivier Bernus, Ruben Coronel, Stanley Nattel and Edward J Vigmond.

Summary: This study develops a detailed model of porcine ventricular cardiomyocyte electrophysiology for the express purpose of understanding how experiments performed with native or in culture pig cardiomyocytes can be translated to predict response in human ventricular cardiomyocytes. Experiments investigating the ion channel I-V relationships and gating in porcine cardiomyocytes from the literature plus some performed by the authors are used to parameterize each ion channel in the model. These parameterized currents are then assembled to represent the qualitative whole cell electrophysiology and calcium handling of a porcine ventricular cardiomyocyte. Then this model is used to explore the generation of early afterdepolarizations and delayed afterdepolarizations with respect to different ion channel blocks. Comparisons are made to similar ion channel blocks in the O'Hara-Rudy model of human ventricular cardiomyocytes. The authors provide a supplement detailing the model equations used and CellML code which can be run in either OpenCOR or JSim which is commendable.

Overall Comments: The model development by the authors is much needed as experiments are now currently being performed investigating the electrophysiological function of porcine cardiomyocytes which need to be translated to function in human cardiomyocytes in order to be useful. The authors make the important note that translation is not straightforward and the different mix of ion channel conductance and function must be considered. The authors fall short of actually suggesting a methodology for this translation although the summary of the differences between the two types of cardiomyocytes is valuable.

The reviewer is correct that the application of the model allows translation of results obtained in pig myocytes to human myocytes. We do not suggest that there is a single methodology for this translation, because multiple ion channels are different between the species Observations made in pig myocardium can easily be related to both models by application of the models.

Specific Major Comments:
1) While developing an electrophysiology and ion handling model to characterize the porcine cardiomyocyte is a necessary first step for translating experimental results from pig to human, besides the observation of differences between these two models no suggestions are made on how to handle the differences for translation. What drugs might be testable in pig cardiomyocytes that would be easy to translate between species? Is the conclusion that the pig is an inadequate model for translation of any arrhythmogenic drug response to humans? Detail in the discussion of this point is lacking.

Response: The authors agree that they could have gone farther in their discussion of ramifications. The conclusion is that drug effects, or any effects, need to be quantitatively identified in the pig model and applied to the human model. The resulting behaviour must then be compared to verify that the findings can be translated. This has now been added to the Discussion (Translation section, pg 12). The section ends with:

'This study highlights differences in calcium handling between the species. Indeed, lack of DADs in porcine models due to application of a drug does not necessarily indicate that the drug is safe in humans. Similarly, arrhythmogenic pathophysiological changes observed in porcine models do not necessarily translate to humans.'

2) It is unclear in the Results, Discussion and Methods sections what experimental results on individual ion channels are taken from Verkerk et al. (Ref 8) and which experimental results were performed by the authors. In the Results, new data is stated to include INa, ICaL, IKr, IKs and IK1 experiments. In the Discussion it appears to be only ICaL, IK1 and IKs and in the Methods ICaL, IK1, IKs and IKr seem to be isolated experimentally

Response: The authors now provide a comprehensive table (Table S1) in the supplement to avoid this confusion.

3) There is little discussion on how the parameters both for the individual ion channel expressions and the porcine ventricular cardiomyocyte model were obtained. The authors should explain clearly what was optimized with the different datasets. A guess from this reader would be that gating expressions and current voltage relationships were fit by hand for each ion channel. Then the whole model was assembled with these ion channels to represent porcine AP and calcium transients with only adjustments by hand to ICaL, IK1 and Ito2 max conductances being made in the model. Were other conductances or parameters optimized at the whole cardiomyocyte model level to match AP and CaT data? If a rigorous optimization was performed what methods were used and would these parameter values be unique?

Response: The author is correct in that gating expressions and IV relationships were fit for the different currents based on experimental data. The whole model then incorporated these new formulations and maximum conductances were set. However, there were many more parameters to set in the model which included more ionic currents than listed by the reviewer, but also physical constants relating to the new Ca release space. The model was fit by hand since it is essentially impossible to fit Ca and voltage simultaneously over many frequencies perfectly. With multiobjective optimization, there is not a single fit, as the particular optimum is based on a particular weighing of the desired outcomes as in a Pareto optimization. The parameters fit and the rationale is now provided in a supplemental table (Table S1).

Specific Minor comments
1) Abstract, line 1: "... including for mechanisms of arrhythmia." Suggest "... including for the study of the mechanisms of arrhythmia."

Response: Done

2) Abstract, general: The authors point out that translation to human myocardium is hampered by the lack of a porcine cardiomyocyte model but the conclusion is to "urge caution" when translating both computational and experimental results across species. Promising less about translation or providing some direction besides urging caution would be two different ways to handle this incongruity between the goals of the paper and the conclusion.

Response: The authors wanted to make the point that humans and pig have points of electrophysiological difference, and that results cannot be blindly translated. With models that capture the differences, we are in a better position to translate effects across species. We have added a section to the discussion on translation and altered the conclusion of the abstract. The conclusion to urge caution has been removed and replaced by a statement about translating results to other species with the help of modelling.

3) Abstract, lines 15-16: Are porcine cardiomyocytes or myocardium resistant to IKr block and sensitive to IK1 block arrhythmogenesis in experiments? If so this should be mentioned in the abstract and the Discussion.

Response: Indeed, swine hearts have been shown to be sensitive to IK1 block (Klein et al, Heart Rhythm, 2017, PMID:28396172) which was included. IKr block by dofetilide did not increase the risk of TdP in swine (Citerni et al, Front Pharmacology, PMID: 32435191, 2020). This reference has been added and both are now referred to in the Discussion, paragraph 3:

In addition, as suggested by our model, pigs have been found to be highly sensitive to ventricular proarrhythmia due to I_{K1} blockade [4] and insensitive to I_{Kr} block [26].

4) Introduction, paragraph 2, line 15: "... results may not be translatable between species.". A stronger statement would be that without computational models to understand the underlying differences between the cardiomyocytes of these two species translating results is not possible.

Response: We appreciate the suggestion and have incorporated it.

5) Results, paragraph 1: More detail about what parameters are adjusted to fit these current-voltage and gating datasets. Maybe a table of the parameters optimized for each ion channel would be helpful. In that table you could also identify where the data is coming from addressing some of major point 2) above. Also in figure 1 each model curve should be plotted as a continuous line instead of the piecewise linear way it is currently depicted.

Response: A comprehensive table has been provided in the supplement (Table S1. More points have been added to the model curves to make them smoother as requested.

6) Results, paragraph 2, line 1: How were these conductances adjusted? Were multiple datasets or just features considered during parameter adjustment Again a table would help here showing everything that was adjusted and what features or time courses were used for the optimization/hand tuning.

Response: A table (Table S1) has been provided to answer these questions. Essentially, the conductances were adjusted by hand.

7) Results, paragraph 2, lines 4-9: Figure 2 referred in this section should have the same time scale on the x axis and Figure 2D should have absolute units of Ca2+ on the y axis with relative units on a second y axis. The experimental data only shows a change in florescence by ~3% so does this translate to a different percentage in absolute Ca2+? It is mentioned in the Methods that this florescence data is from RV samples from pigs. Why RV when everything else was LV?

Response: The authors chose different time scales so that the action potential waveform could be better appreciated. When displayed over a 1000 ms, details are lost, like the initial notch. In figure 2D we now use absolute units. Calibration of optically mapped Ca concentrations is difficult and seldom

performed. The relationship between fluorescence and true $[Ca]_i$ is nonlinear and very sensitive to any parameters assumed. So, a 3% change in fluorescence is a different % change in $[Ca]_i$ since, among other factors, there is a background fluorescence. We used RV since it is the experimental preparation with which we work extensively and its anatomy is more favorable for optical mapping of Ca^{2+}_i. We have noted this in the limitations:

"We used RV preparations from the pig for optical mapping of Ca^{2+} to evaluate model predictions because of its favorable anatomy. For the ion-channel studies for model development, we used pig LV for practical considerations. The qualitative agreement between the model and the RV Ca^{2+} results support the robustness of the model. Further work would be of interest to characterize AP and Ca^{2+} differences between RV and LV, as well as different regions in LV, in order to optimize the model for region-specific differences and determine the mechanisms."

8) Results – Rate-dependence and Restitution of APD section, paragraph 3, line 1: "The pig has lower Ca_NSR, Ca_JSR and Ca_i compared to human.". This should be stated as a prediction made by the simulation since no experimental data is available at this resolution.

Response: The authors have changed the text.

9) Results – Rate-dependence and Restitution of APD section, paragraph 3, line 2: Is the 2mM threshold for humans higher or lower than in O'Hara-Rudy and what warrants a lower threshold in the porcine cardiomyocyte? Discuss what this means here or in the Discussion section.

Response: The ORd model as published, did not incorporate a store-overload induced calcium release (SOICR) mechanism. We ascribed SOCIR thresholds in the models by finding the levels which produced similar DAD behavior for the same pacing protocol. We have indicated this in the text.

10) Results – Rate-dependence and Restitution of APD section, paragraph 3, line 3-8 and then paragraph 4: Are these really DADs? They are not reaching threshold in Figure 7A-D. However when the INa activation curve is shifted by leftward by 10mV DADs do occur (Figure 7E-H). There is no discussion whether this Na shift is physiologically possible. Can a drug do this? Is this just an artifact of the model that DADs can be triggered this way? In figure 7 panels A-D are not necessary.

Response*: We are using the definition of DAD as a depolarization in membrane potential during phase 4 of the action potential, usually due to spontaneous Ca release, whether or not it reaches threshold. As far as the authors are aware, this is the standard definition. We have clarified this in the legends.*

Many class I antiarrhythmic drugs, as well as sodium channel mutations, PMID: 31737628 PMID: 30529471, shift activation curves. This is well known.

We respectfully disagree with the reviewer about figure 7. Figures A-D show DAD that do not lead to triggered activity, whereas figures 7E-H do show triggered activity. This is a significant and clinically relevant difference. Figures 7E-H shows an arrhythmia, panels A-D not. The authors feel that Panels 7A-D are different enough from panels 7E-H to warrant inclusion in the paper.

11) Results – AP and Ca2+ transient alternans section, paragraph 1: This paragraph should be split in two with the first paragraph presenting the experimental results and the second paragraph presenting the simulation results. The simulations are stated to be qualitatively similar to the experiments but there are two noticeable differences: 1) In the simulations at 400 ms cycle length the systolic Ca2+ concentration seems to drop from the 500 ms cycle length and 2) In the simulation the Ca2+ levels in systole and diastole raise dramatically from 300 to 200 ms cycle length. It is made clear that there was no tuning of the model to fit these calcium transients however there should be some acknowledgment

of these differences and discussion of what might be happening in the model and potentially physiologically. Also Figure 8 should have the same time scale on the x axis for panels in A and B. In addition an arrow to show where each higher resolution CaT is pulled from the lower resolution figure would make this figure clearer.

Response: *We have changed Figure 8 to also incorporate the other reviewer's comments. We could not use the exact same time instances for simulation and biology since the biological experiment was performed by hand and was therefore, not constant. We made sure that the time duration depicted in corresponding panels match, however. We added the arrows as suggested. We also added a voltage trace in the last panel to demonstrate voltage alternans. We have discussed the differences in the results as follows: "There are two noticeable differences: 1) at 400 ms cycle length, systolic $[Ca^{2+}]_i$ dropped from the 500 ms cycle length. This is related to the merging of the two phases of the Ca-transient at higher heart rates. 2) Systolic and diastolic $[Ca^{2+}]_i$ rose dramatically from 300 to 200 ms cycle length, although a large rise in diastolic $[Ca^{2+}]_i$ was also seen experimentally, and the Ca-transient duration is well reproduced. Although these likely represent limitations of the model at very high heart rates, it does qualitatively correctly replicate the increase in diastolic Ca-concentration*."

12) Discussion, paragraph 1, lines 5-7: "The model can be used to test potential drug therapies ... ... and allows translation of pig experimental data to human arrhythmogenesis.". This model is the first step to understanding the differences between porcine and human cardiomyocytes but there is no discussion of how knowledge of these differences and the computational model can be used to translate experimental results from porcine myocardium to human myocardium.

Response: A section has been added to the discussion addressing this.

13) Conclusion, paragraph 1, lines 2-6: As to minor point 11) the statement is made that experimental results from porcine myocardium cannot be simply translated to predict function in human myocardium. Can the authors say anything more about an experimental and modeling strategy for translation?

Response: We have modified the comment to be stronger and have incorporated a section on translation.
